# The Role of the Family’s Emotional Climate in the Links between Parent-Adolescent Communication and Adolescent Psychosocial Functioning

**DOI:** 10.1007/s10802-020-00705-9

**Published:** 2020-09-22

**Authors:** Sabina Kapetanovic, Therése Skoog

**Affiliations:** 1 University West, Trollhättan, Sweden; 2 School of Health and Welfare, Jönköping University, Jönköping, Sweden; 3 Gothenburg University, Gothenburg, Sweden

**Keywords:** Family emotional climate, Parent-adolescent communication, Emotional problems, Conduct problems, Wellbeing

## Abstract

The current study was designed to extend the parenting literature by testing the moderating role of the family’s emotional climate, operationalized with parent-adolescent emotional closeness and adolescent feelings of being overly controlled by parents on the longitudinal associations between parent-driven communication efforts (i.e. parental behavioral control and solicitation of information from their adolescent), adolescent-driven communication efforts (i.e. adolescent disclosure and secrecy) and adolescent psychosocial functioning (i.e. emotional problems, conduct problems, delinquency, and wellbeing). We conducted a series of cross-lagged models controlling for adolescent gender and ethnicity using a two-wave Swedish longitudinal set of self-report data (*N* = 1515, 51% girls, M age = 13.0 and 14.3 years at T1 and T2, respectively). Multi-group analyses revealed that the negative links between T1 parental control and T2 adolescent delinquency, T1 parental solicitation and T2 adolescent conduct problems and delinquency, and T1 emotional problems and T2 adolescent disclosure were moderated by the family’s emotional climate. When the family’s emotional climate was positive, the parenting strategies had a more positive effect on adolescent psychosocial functioning, and adolescents with emotional problems communicated more openly with their parents. These findings suggest that the relational context in the family is an important protective factor and add specificity to the previously established role of parent-adolescent communication in adolescent psychosocial development. In terms of preventive interventions, strategies to enhance the family’s emotional climate should be considered prior to teaching specific parenting strategies.

The family’s emotional climate is a key context for adolescent development in general and adolescent-parent communication in particular. The history of interactions between parents and their children, as well as the goals and attributes that parents bring to their parenting socialization, rest within the context of the family’s emotional climate (Darling and Steinberg 1993; Soenens et al. 2019). Embedded within such a context, parents shape their adolescents’ development using different parenting strategies, such as parental behavior control (i.e. communicating behavior control through rules and behavioral expectations) and solicitation (i.e. asking questions of adolescents themselves or by talking to their friends) (Dishion and McMahon 1998; Racz and McMahon 2011). In addition, adolescents manage what parents know about adolescents’ lives with their own communication efforts, including adolescent disclosure (i.e. sharing information about their everyday activities with their parents) (Kerr and Stattin 2000) and adolescent secrecy (i.e. withholding information from parents) (Finkenauer et al. 2002). The idea is that with the help of these parent-driven and adolescent-driven communication efforts, parents can promote positive developmental outcomes for their adolescents and protect them from harm. Parent-driven and adolescent-driven communication efforts are however embedded within the history of parent-child interactions which shape adolescent perception of the family context. In that sense, the question that emerges is whether parent-driven and adolescent-driven communication efforts can be effective in terms of adolescent development in some contexts but not in others. To answer this question, the current study will investigate the moderating role of adolescents’ perceptions of the family emotional climate, operationalized with adolescents’ perceptions of parent-adolescent emotional closeness and adolescents’ feelings of being overly controlled by parents, in the longitudinal associations between parent-driven communication efforts (i.e. parent control and solicitation), adolescent-driven communication efforts (i.e. adolescent disclosure and secrecy) and adolescent psychosocial functioning (i.e. emotional problems, conduct problems, delinquency and wellbeing).

Over a number of decades, parenting has been depicted through the lens of Baumrind’s typologies of parenting authority and styles (Maccoby and Martin 1983) with the idea that the parenting dimensions characterized by different constellations of parenting practices, attitudes toward the child, and the assertion of power by parents (i.e. authoritative, authoritarian, neglecting, and permissive styles) constitute the core-base of children’s socialization and psychosocial development. In that sense, parents shape their children’s development by using their power and authority over their children. However, the way that parents use their power in parenting has differential effects on children’s psychosocial development. Accordingly, when parents express their authority with demandingness and responsiveness toward their children, their children’s development is expected to be more promising than that of children whose parents express their authority with coercive and harsh assertion of power toward their children (Baumrind et al. 2010). As an extension of this idea, Smetana et al. (2006) proposed that adolescents need to perceive parental authority as legitimate if parents’ expressions of power are to have any beneficial effect on adolescent psychosocial development (Smetana 2017). In this way, both parents and their adolescent children actively shape adolescent psychosocial development.

According to the developmental contextual view on parenting (Lerner et al. 2002), adolescent development happens in interaction with different social contexts, where parents often play the most prominent role. Parents and their children are part of different social and cultural systems in which they mutually influence the development of their parent-child relationships and bonds. Accordingly, parents bring their cognitions, attitudes, and goals to their parenting, which guides them in the socialization of their children. Although parenting literature often focuses on parent-to-child effects, parenting does not stand independent from children’s effect on their parents. Thus, in the developmental contextualism view of parenting, children influence parents with behavioral and emotional reactions to their parents. In this way, they have an effect on the parenting style used by parents (Kerr et al. 2012). As it seems, parents’ expression of authority and assertion of power operates within a family context, where parents and children mutually affect each other with their behaviors, attitudes, and goals. Thus, such context encompasses family processes in which both parents and their children are involved. One such process is the family’s emotional climate.

In their integrative model of parenting, Darling and Steinberg (1993) proposed that parental expression of authority and thus, parenting style, rests within the context of the family’s emotional climate. In this context, parents’ attitudes toward the child are communicated to the child, creating an emotional climate in which parents and children interact. Accordingly, and in line with Lerner et al. (2002), the family’s emotional climate is in part influenced by parents’ socialization goals and values. In addition, a family’s emotional climate is also influenced by the history of interactions between parents and their children. For example, from early stages of life, parents and their children form emotional bonds to each other, which often persist during middle childhood and adolescence (Jones et al. 2018). Such an emotional bond between parents and their children is a core-base for the quality of the parent-child relationship and children’s perception of parents’ behaviors and goals. In essence, the family’s emotional climate could be seen as a constellation of parent-child emotional bonds, parenting attitudes, and the child’s perception of parenting behaviors.

When a family’s emotional climate is positive, the parenting strategies that parents use to socialize their children may have more promising effects on the child’s development. Parenting strategies are the discrete mechanisms through which parents guide their children to attain socialization goals set by parents. These strategies operate within the context of the family’s emotional climate. For example, growing up within the walls of a family where there is a positive emotional climate, including parent-child closeness (Kapetanovic et al. 2019b) and warmth (Lansford et al. 2018) could enhance children’s openness to being socialized by their parents. If children are open to socialization, parents may have the opportunity to promote positive developmental outcomes and protect their children from harm through the use of adequate parenting strategies. On the other hand, if the context of the family’s emotional climate is poor, the parenting strategies used by parents could have little or even a harmful effect on a child’s developmental outcomes (Darling and Steinberg 1993). Therefore, whether or not parents’ strategies would have a promising effect on children’s psychosocial functioning may depend on the context of the family’s emotional climate.

Focusing on discrete dimensions of parenting, such as parenting strategies, provides a more specific understanding of the processes and mechanisms operating within a family. Indeed, parents’ active efforts in parenting have long been regarded as key factors for preventing adolescents from engaging in risk behaviors and for promoting positive adolescent psychosocial development. Such active efforts include parent-driven communication strategies, such as parental solicitation (i.e. asking questions of adolescents themselves, or by talking to their adolescents’ friends and parents of their friends in order to obtain information of their adolescents’ whereabouts) and parental behavioral control (i.e. communicating rules of behavior and controlling adolescents’ freedom to come and go as they please; Stattin and Kerr 2000), also referred to as parental monitoring (Dishion and McMahon 1998). The idea is that parents who ask questions of adolescents, impose rules, and have behavioral expectations are more involved in their adolescents’ lives, which in turn makes it possible for them to protect their adolescents from maladjustment. Indeed, empirical research provides support for such an idea, showing that parent-driven communication efforts protect against the development of externalizing (Pinquart 2017a) and internalizing problems (Pinquart 2017b) as well as delinquency (Hoeve et al. 2009) in adolescence.

Although parents are regularly seen as key figures in their children’s socialization, children are not passive, but are themselves active in their own psychosocial development and interaction with parents (Lerner et al. 2002). Indeed, Kerr and Stattin (2000) brought attention to the notion of the active child and children’s information management within a family by including the voluntary sharing of information by adolescents (i.e. disclosure) as a central part of parent-adolescent communication and adolescent psychosocial development. They found that when adolescents shared information about their everyday activities with their parents, parents could impose certain strategies to protect their adolescent children from maladjustment. Moreover, Kerr and Stattin suggested that adolescent-driven efforts are central for parent-adolescent interactions and adolescent development, above and beyond parents’ own communication efforts. Other more recent studies provide support for such ideas, showing that adolescent disclosure is linked to less delinquency (Kapetanovic et al. 2019a), externalizing problems (Racz and McMahon 2011) and internalizing problems (Fernandez et al. 2018) over time. When adolescents share information with their parents, they inevitably allow more involvement from their parents. When parents are involved and have information about their adolescents’ activities, they have the possibility to provide guidance and support and, in this way, protect their adolescents from negative developmental outcomes.

Another way for adolescents to manage how much information their parents receive is to withhold it from their parents. One of the reasons for adolescents’ withholding information from parents could be part of normative development in adolescence. Withholding information is used by adolescents as means to liberate themselves from parents in the adolescent struggle for autonomy and independence (Finkenauer et al. 2002). Another reason for adolescents to withhold information from parents is because they know that they have engaged in behaviors that parents would not approve of (Marshall et al. 2005). Either way, adolescent information withholding is a communication process that leaves parents with less information about their adolescents’ whereabouts, and thus with fewer possibilities to guide their adolescents in their development. Thus, disclosure and secrecy should also be seen as two separate factors in parent-adolescent communication that uniquely contribute to adolescent psychosocial development (Frijns et al. 2010). In addition, adolescent secrecy seems to be related to higher levels of depression (Frijns et al. 2010) and delinquency (Jäggi et al. 2016) and poorer wellbeing (Elsharnouby and Dost-Gözkan 2020). Moreover, a recent cross-cultural study by Kapetanovic et al. (2020) shows that adolescent secrecy, but not disclosure, is reciprocally linked to higher levels of externalizing problems over time. Given these recent findings, in this study we will treat adolescent driven communication as two separate constructs as they pertain to adolescent psychosocial functioning.

Seen from the review above, parenting processes are complex and include different aspects of parenting attributions, where the context of a family’s emotional climate and parenting strategies play important roles. Parents’ expressing demands like “You need to tell me where you are going” and asking questions such as “Where have you been?” can be perceived by the adolescent either as a sign of genuine concern (Kapetanovic et al. 2019a) or as an invasion of privacy (Hawk et al. 2008). How adolescents perceive such parenting efforts may be reflected in the context of the family’s emotional climate. In families where parental warmth is lacking (LaFleur et al. 2016) adolescents often interpret parents’ controlling efforts as overly controlling, and perceive parents as compromising their autonomy and need for privacy (Kakihara et al. 2010). In such a family context, parental communication efforts are linked to poorer developmental outcomes (Hessel et al. 2017). On the other hand, adolescents whose parents convey behavioral expectations and demands within a context of the family’s positive emotional climate, have more promising psychological outcomes than adolescents whose parents are psychologically controlling and thus, unpredictable and intrusive (Rodríguez-Meirinhos et al. 2020). Moreover, when adolescents feel emotionally connected to their parents, they are likely to disclose information to their parents; however, when adolescents feel overly controlled by their parents, they are more likely to withhold information about their whereabouts and activities (Tilton-Weaver et al. 2010). As a consequence of adolescent disclosure or secrecy, adolescent psychological development may be more or less promising (Kapetanovic et al. 2020; Marshall et al. 2005). As it seems, the emotional climate perceived by the adolescent could be an underlying structure that facilitates or impedes adolescent-driven communication with parents and its effect on adolescent psychological outcomes.

Mutual parent-child actions, parenting attributions and goals constitute the family’s emotional climate in which parents’ attitudes toward the child, rather than toward the child’s behavior, are expressed. Thus, strategies that parents use to promote their children’s positive development or prevent negative developmental outcomes, do not operate unrelated to the family’s emotional climate (Darling and Steinberg 1993). As an extension to the parenting literature, this two-wave longitudinal study examined the moderating role of adolescents’ perceptions of the family’s emotional climate, operationalized with parent-adolescent emotional closeness and adolescent feelings of being overly controlled by parents, on the links between parent-driven communication efforts (i.e. parental behavioral control and solicitation), adolescent-driven communication efforts (i.e. adolescent disclosure and secrecy) and adolescent psychosocial functioning (i.e. emotional problems, conduct problems, delinquency and wellbeing). Guided by parenting theories (e.g. Darling and Steinberg 1993) and research (e.g. Hoeve et al. 2009; Kapetanovic et al. 2019a), we expected (a) that parent-driven communication efforts (i.e. parental solicitation and behavioral control) and adolescent disclosure would be associated with less externalizing problems, internalizing problems, and delinquency and more wellbeing over time, and (b) that adolescents’ perceptions of the family’s emotional climate would have a moderating role in the links between parent-driven communication and adolescent psychosocial functioning such that parent-driven communication would be linked to less adolescent psychosocial problems (i.e. externalizing, internalizing and delinquency) and more wellbeing, only within the context of a high family emotional climate. Given that adolescents share information when parents are emotionally connected to them (Kapetanovic et al. 2019b), but that adolescents withhold information when they perceive that they are being overly controlled by their parents (Tilton-Weaver et al. 2010), we expected that adolescent disclosure would be related to less psychosocial problems in the context of high family emotional climate and that adolescent secrecy would be related to more psychosocial problems in the context of low family emotional climate.

## Method

### Participants

Our data come from a Swedish research program, Longitudinal Research on Development in Adolescence (LoRDIA; Kapetanovic et al. 2019a). The program is designed to follow adolescents in four medium-sized municipalities in southern Sweden for 4–5 years, from 12/13 to 18 years of age. Out of a total of 2150 invited students in Wave 1, 6.6% opted out, which resulted in 1780 adolescents, constituting the total sample of the study at Wave 1. A total of 6.7% of the adolescents were absent from school on the days of the data collection, which resulted in an analytical sample of 1515 adolescents. The measures used in LoRDIA vary somewhat between waves. This means that some data were only collected once or twice.

The sample for the current study is based on two waves (from now on referred to as T1 and T2) of self-reported data with *N* = 1515 adolescents (50.6% girls) beginning in sixth grade (*n* = 781) and seventh grade (*n* = 734), respectively. The mean ages were T1: *M* = 13.01 years (*SD* = 0.60); T2: *M* = 14.33 years (*SD* = 0.64). Most lived with both parents (80.6%) and were of Swedish ethnicity (80.5%). Nearly 20% were persons of other Western origin, Middle East or Africa. Most of the adolescents (62.8%) reported having as much money as their classmates, while 20.3% reported that their family had more money than their classmates’ families, and 16.8% reported that their family had less money than the families of their classmates. The participants included at T1 and those who opted out were compared using available register data on demographics (gender and immigration status) and school performance (absenteeism and merit points based on grades) to assess the representativeness of the sample used. There were no significant differences in gender (*p* = 0.22), immigrant status (*p* = 0.07), merit points (*p* = 0.15), or absence from school (*p* = 0.60) which indicates that the sample is representative for the target sample based on gender, immigrant status, and school performance.

### Procedure

In 2013, we established contact with all middle schools in the participating municipalities and with the parents of the students. Students, as well as their parents, were sent letters with information about the study. The letter, translated into 32 different languages, explained the nature of the survey, and informed about opt-out parental consent, where the parents were given the opportunity to decline their child’s participation. The students replied annually to questionnaires, which were collected in the classrooms by the research team. The study received ethical approval from the Regional Research Review Board in Gothenburg before each data collection wave.

### Measures

#### Parent-Adolescent Communication: Parental Behavioral Control, Solicitation, Adolescent Disclosure and Secrecy

The measure frequently used in studies on parent-adolescent communication (e.g. Frijns et al. 2010; Kapetanovic et al. 2020) come from Stattin and Kerr (2000). Parental behavioral control assessed ways in which parents set rules and regulations to control and regulate adolescents’ behavior. The measure was based on five items, such as “Do you need to have your parents’ permission to stay out late on a weekday evening?”. Parental solicitation measured how often parents initiate conversation with their adolescent with five items such as “How often do your parents ask you what happened during your free time?” Adolescent disclosure assessed how often adolescents share information with their parents with three items such as “Do you like to tell your parents where you went and what you did during the evening?” Adolescent secrecy assessed how often adolescents kept secrets from their parents with two items such as “Do you hide a lot from your parents about what you do during nights and weekends?” (Frijns et al. 2010). Higher scores indicate greater parental behavioral control and solicitation and adolescent disclosure and secrecy. The ratings ranged from 1 (*Never*) to 3 (*Always*). Measure has proven to be internally consistent in samples of adolescents with alphas ranging from 0.64 to 0.82 (Lionetti et al. 2016).

#### Adolescent Emotional Problems and Conduct Problems

Two out of five subscales in the Swedish self-report version of the Strength and Difficulties Questionnaire (SDQ-S) were used to assess adolescent emotional problems and conduct problems (Lundh et al. 2008). Adolescent emotional problems were assessed with five items, including “I worry a lot,” “I get a lot of headaches, stomach aches or sickness,” “I am often unhappy, down-hearted or tearful,” “I am nervous in new situations, I easily lose confidence,” and “I have many fears, I am easily scared.” Adolescent conduct problems were assessed using five items, including “I get very angry and often lose my temper,” “I usually do as I am told” (rev), “I fight a lot. I can make other people do as I want,” “I am often accused of lying or cheating,” and “I take things that are not mine from home, school or elsewhere.” The ratings ranged from 0 (*Not true*) to 2 (*Completely true*). Although alpha for the conduct problems scale was poor (α = 0.48), the measure has been proven to have good test-retest reliability and predictive validity (Lundh et al. 2008).

#### Adolescent Delinquency

The measure was taken from the annual school survey conducted by The Swedish National Council for Crime Prevention (Ring 2013) and assessed adolescent engagement in delinquent behaviors with five items, such as “How many times have you stolen something from a shop?” and “How many times have you threatened someone to get that person’s money or other belongings?” The ratings ranged from 1 (*Never*) to 3 (*Several times*). The measure has been proven to be internally consistent with alphas ranging from 0.67 to 0.79.

#### Adolescent Wellbeing

This measure assessed adolescents’ life satisfaction, as well as their sense of purpose and meaning in life (Berlin et al. 2012). It consisted of two questions: “In general, how satisfied are you with your life at the moment?” with ratings ranging from 1 (V*ery happy*) to 4 (*Very unhappy*), and “I think that my life has purpose and meaning” with ratings ranging from 1 (*Completely agree*) to 4 (*Completely disagree*). The responses were later reversed so that higher values indicated higher levels of wellbeing. The measure has been proven to be internally consistent with Spearman Brown coefficients ranging from 0.73 to 0.75. The items used are similar to items measuring subjective and psychological well-being in the Mental Health Continuum Short Form (Keyes 2009).

#### Demographics

Adolescent gender and ethnicity at T1 were included in analyses as covariates and predictors of T2 adolescent psychological outcomes. Adolescent gender was entered as “1” for female and “2” for male. Ethnicity was determined by asking the adolescents if they studied Swedish as a second language in school and entered as 0 = Swedish ethnicity and 1 = Non-Swedish ethnicity.

To provide a quantitative sense of the effects of adolescents’ perceptions of the family’s emotional climate on adolescent psychosocial outcomes, we tapped into aspects of family dynamics similar to what has been used and described in previous research (Morris et al. 2007; Woodman et al. 2015). The following measures were dichotomized and used as grouping variables in multi-group analyses. A median split was used to dichotomize scores into high and low scores. The moderating measures were collected at Time 1 (T1).

#### Feeling Connected to Parents

The scale, developed by Biesecker (2007) (and used in Tilton-Weaver et al. 2010) measured to what extent adolescents felt emotional connectedness to their mothers and fathers respectively, using nine items such as “When I am angry, sad, or worried, my mother/father can make me feel better.” The ratings ranged from 1 (*Not true*) to 3 (*Completely true*) because reports for mothers and fathers were substantially correlated (r (1464) = 0.75, *p* < 0.01) and subsequently averaged. The measure has been proven to be internally consistent in samples of adolescents with alphas ranging from 0.85 to 0.91.

#### Adolescent Feelings of Being Overly Controlled

This scale, developed by Kerr and Stattin (2000) (and used in Tilton-Weaver et al. 2010) measured how controlled adolescents felt regarding parental behavioral control with five items such as “Does it feel like you can’t keep anything to yourself, because your parents want to know everything?” The ratings ranged from 1 (*Not true*) to 3 (*Completely true*). The measure has been proven to be internally consistent in samples of adolescents with alphas ranging from 0.69 to 0.82 and good test-retest reliability (Kerr and Stattin 2000).

### Measurement Invariance, Missing Data Analysis and Attrition

Before proceeding with the analyses, we estimated iterative series of confirmatory factor analyses (CFAs) to confirm the internal structure of the scales during T1 and T2. All factors provided an acceptable model fit indicated by CFI > 0.90, TLI > 0.95 and RMSEA <0.08. After the CFAs were completed, for each measure we compared models with unconstrained and constrained factor loadings over time in order to test measurement invariance. The relative fit of the constrained model was evaluated based on change in CFI (ΔCFI). The change in each model was <0.01 which indicated an equivalent fit between the models (Van de Schoot et al. 2012).

We tested whether the missing data was missing at random. Missing data analysis showed that Little’s MCAR (Missing Completely at Random) was significant, however the normed chi-square (χ2/df) was low (1509.587/1103 = 1.37), implying a low violation of the MCAR assumption. Further attrition analyses showed that 75.3% of the original sample (*N* = 1515) continued to provide data at T2. The attrited adolescents reported lower levels of adolescent disclosure (MAttrited = 2.35, MRetained = 2.42, *p* = 0.032, *d* = 0.14) and higher levels of conduct problems (MAttrited = 0.46, MRetained = 0.32, *p* = 0.002, *d* = 0.17) at baseline. Attrited adolescents did not significantly differ from the retained adolescents in terms of gender, ethnicity, parent-driven communication efforts, adolescent secrecy, feelings of being overly controlled, parent-adolescent emotional connectedness, emotional problems, delinquency, or wellbeing at baseline. As Cohen’s d effect sizes were small (0.20 can be interpreted as small effect, 0.50 medium effect and 0.80 as large effect (Cohen 1992)), we included all variables in analyses and utilized full information maximum likelihood (FIML) estimation procedure to account for missing data. With FIML, it is possible to produce unbiased parameter estimates and bias-corrected confidence intervals (Byrne 2010).

### Data Analysis

We calculated bivariate correlations between the study constructs. Subsequently, we implemented structural equation modelling using AMOS 23.0 in four steps. First, we fitted separate measurement models to evaluate autoregressive cross-lagged associations between each parent-adolescent communication measure (i.e. parental behavioral control and solicitation and adolescent disclosure and secrecy) and each outcome measure (i.e. adolescent emotional problems, conduct problems and wellbeing). Thus, the latent constructs of each T1 parent-adolescent communication measure were regressed on the latent constructs of each T2 adolescent psychological health measure, and the latent constructs of each T1 adolescent psychological health measure were regressed on each T2 parent-adolescent communication measure. Next, to obtain the most parsimonious model, in the final model we constrained the factor loadings in the constructs to be the same across time points. Evaluation of model fit was based on recommended fit index cut-off values (CFI > 0.90, TLI > 0.95, RMSEA <0.08 (Byrne 2010).

Finally, we conducted an iterative series of multi-group analyses to test whether the links between the latent constructs were moderated by family emotional climate, measured by adolescent connectedness to parents and feelings of being overly controlled by parents. A constrained model, where effects are equivalent across groups, was compared with an unconstrained model with freely varying effects using χ^2^-difference tests. A significantly better fit of the unconstrained model (as indicated by significant Δχ^2^) would indicate a moderation effect (Byrne 2010).

## Results

### Links between Parent-Adolescent Communication and Adolescent Psychosocial Functioning

**Fig. 1:**
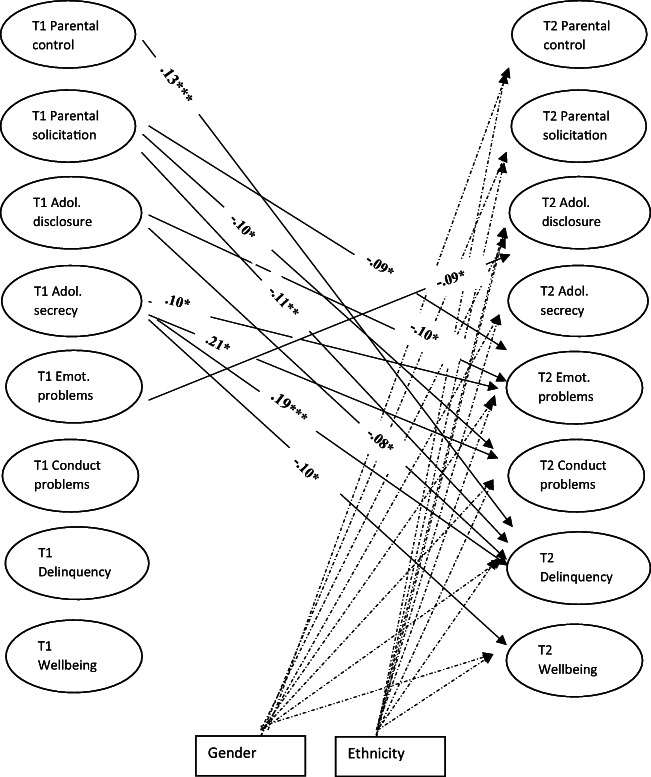
Cross-lagged links between parent-adolescent communication efforts and adolescent psychological health and delinquency over time controlling for gender and ethnicity. Note: *<0.05 **<0.001 *Adol*. Adolescent, *Emot.* Emotional

Table 1 provides the means, standard deviations, and correlations among the study’s variables. All bivariate final models had acceptable model fit (Table 2). As shown in Fig. 1, T1 parental behavioral control (β = 0.13 *p* < 0.001) was positively linked to T2 adolescent delinquency, while T1 parental solicitation was negatively linked to T2 adolescent emotional problems (β = −0.09 *p* = 0.001), T2 adolescent conduct problems (β = −0.10 *p* = 0.009) and T2 adolescent delinquency (β = −0.11 *p* < 0.001). T1 adolescent disclosure was negatively linked to T2 emotional problems (β = −0.12 *p* = 0.012) and T2 adolescent delinquency (β = −0.08 *p* = 0.011). T1 adolescent secrecy was positively associated with T2 adolescent emotional problems (β = 0.10 *p* < 0.001), T2 adolescent conduct problems (β = 0.21 p < 0.001) and T2 adolescent delinquency (β = 0.19 p < 0.001) and negatively linked to T2 adolescent wellbeing (β = 0.10 p = 0.012). T1 adolescent emotional problems were negatively linked to T2 adolescent disclosure (β = −0.08 *p* = 0.015).

**Table 1:** Means, standard deviations, and correlations among study variables

	1	2	3	4	5	6	7	8	9	10	11	12	13	14	15	16	17	18	19	20
1. Gender	–																			
2. Ethnicity	0.02	–																		
3. T1 PAC	−0.02	−0.05*	–																	
4. T1 FBOC	0.01	0.07**	−0.11**	–																
5. T1 Control	−0.13**	−0.02	0.13**	0.31**	–															
6. T1 Solicit	−0.15**	−0.03	0.33**	0.14**	0.31**	–														
7. T1 Discl	−0.15**	−0.04	0.47**	−0.04	0.26**	0.56**	–													
8. T1 Secrecy	0.08**	0.01	−0.36**	0.16**	−0.15**	−0.20**	−0.43**	–												
9. T1 Conduct	0.15**	−0.02	−0.29**	0.16**	−0.13**	−0.15**	−0.32**	0.35**	–											
10. T1 Emotion	−21**	−0.01	−0.24**	0.16**	0.10**	−0.06**	−0.11**	0.16**	0.28**	–										
11. T1 Delinq	0.12**	−0.03	−0.29**	0.04	−0.12**	−0.07**	−0.23**	0.25**	0.34**	0.10**	–									
12. T1 Well	0.17**	0.01	0.43**	−0.13*	−0.03	0.20**	0.31**	−0.26**	−0.28**	−0.47**	−0.16**	–								
13. T2 Control	−0.13**	0.01	0.07*	0.20**	0.40**	0.16**	0.15**	−0.11**	−0.07*	0.04	−0.05	0.05	–							
14. T2 Solicit	−0.18**	−11**	0.26**	0.04	0.22**	0.47**	0.38**	−0.17**	−0.16**	−0.02	−0.06*	0.16**	0.22**	–						
15. T2 Discl	−0.15**	−0.12**	0.36**	−0.04**	0.18**	0.39**	0.49**	−0.31**	−0.24**	−0.07	−0.15**	0.25**	0.22**	0.55**	–					
16. T2 Secrecy	0.09**	0.02	−0.17**	0.09**	−0.10**	−0.14**	−0.25**	0.34**	0.17**	0.08**	0.15**	−0.12**	−0.10	−0.11**	−0.39**	–				
17. T2 Conduct	0.09**	0.02	−0.21**	0.09**	−0.11**	−0.13**	−0.25**	0.32**	0.40**	0.15**	0.32**	−0.18**	−0.18**	−0.19**	−0.34**	0.39**	–			
18. T2 Emotion	−0.38**	0.05	−0.17**	0.12**	0.05	−0.06	−0.08**	0.14**	0.14**	0.53**	0.03	−0.40**	0.08**	−0.06*	−0.12**	0.14**	0.25**	–		
19. T2 Delinq	0.18**	−0.01	−0.15**	0.03	−0.15**	−0.12**	−0.19**	0.26**	0.24**	0.03	0.47**	−0.08**	0.19**	−0.12**	−0.28**	0.32**	0.42**	0.01	–	
20. T2 Wellb	0.23**	−0.02	0.23**	−0.08**	0.01	0.14**	0.19**	−0.20**	−0.17**	−0.34**	−0.11**	0.46**	−0.01	0.18**	0.29**	−0.24**	−0.27**	−0.52**	−0.09**	–
Mean	–	–	2.66	1.56	2.21	2.16	2.41	1.38	0.37	0.52	1.04	2.41	2.13	2.18	2.27	1.44	0.35	0.63	1.08	2.36
SD	–	–	0.42	0.51	0.55	0.48	0.55	0.54	0.29	0.42	0.15	0.58	0.56	0.44	0.54	0.54	0.30	0.49	0.23	0.60

***Note: <0.05 <0.001; *PAC* Parent-adolescent connectedness, *FBOC* Feelings of being overly controlled, *Solicit* Solicitation, *Discl* Disclosure, *Conduct* Conduct problems, *Emotion* Emotional problems, *Delinq* Delinquency, *Wellb* Wellbeing

**Table 2:** Model fit indices for all models

	χ^2^	*df*	CFI	TLI	RMSEA
Model					
Control – Conduct problems	554.82	190	0.95	0.94	0.04
Control – Emotional problems	518.82	189	0.95	0.93	0.03
Control – Delinquency	549.63	189	0.96	0.95	0.03
Control – Wellbeing	219.11	88	0.97	0.96	0.03
Solicitation – Conduct problems	562.47	190	0.94	0.92	0.04
Solicitation – Emotional problems	562.95	190	0.95	0.95	0.04
Solicitation – Delinquency	565.17	187	0.95	0.94	0.04
Solicitation – Wellbeing	301.18	83	0.96	0.95	0.04
Disclosure – Conduct problems	312.53	112	0.95	0.94	0.03
Disclosure – Emotional problems	362.59	119	0.96	0.95	0.04
Disclosure – Delinquency	440.30	120	0.95	0.94	0.04
Disclosure – Wellbeing	119.02	43	0.98	0.96	0.03
Secrecy – Conduct problems	255.99	86	0.95	0.93	0.04
Secrecy – Emotional problems	348.75	89	0.95	0.94	0.04
Secrecy – Delinquency	351.58	87	0.96	0.94	0.04
Secrecy – Wellbeing	42.76	20	0.99	0.98	0.03

### The Role of the Family’s Emotional Climate

For each of the bivariate models we applied separate multi-group analyses to test the moderation brought about by adolescent connectedness to parents and feelings of being overly controlled by parents, as measures of a family’s emotional climate, on the links between parent-adolescent communication efforts and adolescent psychosocial functioning. The moderation by the family’s emotional climate emerged in links between T1 parental solicitation and T2 adolescent conduct problems, T1 parental behavioral control and T2 adolescent delinquency, T1 parental solicitation and T2 adolescent delinquency and T1 adolescent emotional problems and T2 disclosure. T1 parental solicitation was linked to lower levels of T2 adolescent conduct problems (Δχ^2^ (1) = 4.21, *p* = 0.040) only in adolescents who rated their feelings of being overly controlled by parents as low (β = −0.16 p = 0.011) compared to those who rated their feelings of being overly controlled by parents as high (β = −0.01 *p* = 0.883). Moreover, T1 parental behavioral control was linked to higher levels of T2 adolescent delinquency (Δχ^2^ (1) = 10.59, *p* = 0.001) only in adolescents who rated their connectedness to their parents (β = 0.21 p < 0.001) as low compared to those who rated their connectedness to their parents as high (β = 0.01 *p* = 0.789). T1 parental solicitation was linked to lower levels of T2 adolescent delinquency (Δχ^2^ (1) = 4.61, *p* = 0.032) only in adolescents who rated their feelings of being overly controlled by parents as low (β = −0.19 p < 0.001) in contrast to those who rated their feelings of being overly controlled by their parents as high (β = −0.07 *p* = 0.184). Finally, T1 adolescent emotional problems were linked to lower levels of T2 adolescent disclosure (Δχ^2^ (1) = 6.55, *p* = 0.010) only in adolescents who rated their connectedness to their parents as low (β = −0.19 p = 0.010) in contrast to those who rated their connectedness to their parents as high (β = 0.07 *p* = 0.229).

## Discussion

Interactions between parents and their adolescent children rest within the context of the family’s emotional climate. Parents and children mutually affect each other with their behaviors, attitudes, and goals, creating a foundation of emotional climate in families where parents and their children interact (Soenens et al. 2019). Adolescents’ perceptions of the family’s emotional climate shapes adolescents’ meaning making of their interactions with their parents. For instance, adolescents interpret the general affective tone of the parent-adolescent interactions, which in turn affects how they interpret other parental behaviors and also how they choose to behave themselves. This study extends our understanding of the role played by a family’s emotional climate – in the perspective of the adolescent – for the longitudinal associations between parent-driven communication efforts (i.e. parental behavioral control and solicitation), adolescent-driven communication efforts (i.e. adolescent disclosure and secrecy) and adolescent psychosocial functioning (i.e. emotional problems, conduct problems, delinquency and wellbeing). The findings from a series of bivariate cross-lagged models with multi-level analyses revealed that the positive link between parental control at time 1 and adolescent delinquency at time 2, the negative links between parental solicitation at time 1 and adolescent conduct problems and delinquency at time 2, as well as the negative link between adolescent emotional problems at time 1 and adolescent disclosure at time 2, were moderated by the family’s emotional climate. Seen in the perspective of the adolescent, the family’s emotional climate sets a foundation for parent-child communication and its developmental sequels.

Parent-adolescent communication is a key aspect of parent-adolescent relationships and is generally protective of adolescent psychological functioning (Soenens et al. 2019). We found that parent-driven communication efforts (i.e. solicitation and behavioral control) were predictive of the adolescent’s externalizing problems (i.e. conduct problems and delinquency), such that parental solicitation was linked to lower levels of adolescent conduct problems and delinquency over time, while parental behavioral control was linked to higher levels of adolescent delinquency over time. These links were moderated by the level of the family’s emotional climate. Adolescents have specific perceptions of how their parents make them feel and what attitudes their parents have toward them which ultimately constitute adolescent perceptions of the family’s emotional climate. Depending on the perceived quality of such a family context, adolescents could be more or less open to parents’ socialization. Specifically, in the context of a family’s positive emotional climate, parents’ questions and requests for information turned out to be protective against the adolescent’s externalizing problems. In contrast, parental solicitation did not show any protective longitudinal effects on adolescent externalizing problems in the context of a family’s negative emotional climate. These findings are helpful for understanding the somewhat inconsistent findings in the literature concerning links between parental solicitation and adolescent psychosocial outcomes, where some studies find protective effects (Laird et al. 2010), and other studies show non-significant (Bendezú et al. 2018) or even negative (Hessel et al. 2017) effects of parental solicitation on adolescent psychosocial outcomes. How adolescents perceive and make meaning of the general emotional climate in the family seems to be important for the effect that parents’ actions may have on adolescent outcomes. It is thus possible that in the context of a family’s positive emotional climate, adolescents perceive their parents’ questions about their whereabouts as signs of love and caring (Brown and Bakken 2011). In the context of a family’s poor emotional climate, the same practices might be perceived as intrusive (Hawk et al. 2008) and therefore have disadvantageous effects on adolescent psychological functioning.

Furthermore, the positive link between parental behavioral control and adolescent delinquency was found only in families with a poor emotional climate. One explanation of such findings concerns adolescents’ beliefs about the legitimacy of parental authority (Smetana and Rote 2019). As children make the transition to adolescence, their need for autonomy and jurisdiction over personal issues grows. Parents, on the other hand, need to adjust their parenting practices to be able to effectively socialize and protect their children while allowing for more child autonomy. Possibly, in the context of a family’s poor emotional climate, parents are unable adequately to adjust their parenting practice to match their adolescent’s autonomy demands. As a result, adolescents could perceive their parents’ rules as illegitimate and overly controlling. This, in turn, may trigger adolescent frustration and mistrust toward parents (Van Petegem et al. 2020) manifesting in greater psychosocial problems in adolescents (Rodríguez-Meirinhos et al. 2020).

In addition, we found that parental solicitation was negatively linked to adolescent emotional problems over time, suggesting that parents’ questions about an adolescent’s whereabouts could be protective against the development of adolescent emotional problems. The effect size was however small (β = −0.09) and should therefore be interpreted with caution. This link was not moderated by the family’s emotional climate. Thus, whereas a family’s emotional climate moderated the links between parents’ actions and adolescent externalizing problems, this was not the case regarding adolescent internalizing problems. A similar result was shown in a study by Formoso et al. (2000), who found that parent-adolescent attachment moderated the link between family conflict and adolescent conduct problems, but not the link between family conflict and adolescent depression. It is possible that parents’ showing of interest in their child’s activities could have some effect on adolescent emotional problems regardless of the level of the family’s emotional climate. Parents’ asking questions about adolescents’ activities as well as about their relationships with friends, could be perceived by adolescents as an attempt to be involved in adolescents’ lives. When perceived as such, parental solicitation elicits disclosure in adolescents (Baudat et al. 2020), which in turn gives parents opportunities to provide support and ease adolescent emotional distress. Indeed, parental involvement is linked to improved self-esteem and emotional self-regulation skills, which in turn are protective against the development of depressive symptoms (Flouri and Buchanan 2003). Thus, when parents ask questions and show interest in their adolescents’ lives, that may give parents opportunities to help their adolescents to cope with emotional difficulties. In that way parents may help to reduce the risk of the adolescent developing emotional problems.

Corroborating results from other studies, we found that adolescent disclosure was linked to less adolescent emotional problems (Fernandez et al. 2018) and delinquency (Kapetanovic et al. 2019a) over time, while adolescent secrecy was linked to more emotional and conduct problems (Kapetanovic et al. 2020), delinquency (Frijns et al. 2010), and poorer wellbeing (Elsharnouby and Dost-Gözkan 2020) over time. In addition, higher levels of adolescent emotional problems predicted less disclosure over time, demonstrating the reciprocal nature of these links. None of the links from adolescent-driven communication efforts to adolescent psychosocial problems were moderated by the family’s emotional climate. Although past research indicates that parent-adolescent connectedness (Tilton-Weaver et al. 2010), a facet of a family’s emotional climate, affects adolescent information management, what actually explains the link between adolescent communication efforts and psychological functioning is a puzzle that needs more exploration. Adolescent communication-efforts are an adolescent-induced mechanism, and it is therefore possible that other underlying processes or mediating mechanisms drive the associations between adolescent communication efforts and adolescent psychological functioning. In other words, adolescent disclosure is a process with cognitive, affective, and behavioral components. Adolescents first need to decide whether to share or withhold information from their parents, why they would do it, and then act accordingly. As such, it is possible that adolescent cognitive processes could play a role. Although parents’ expectations and attitudes are indeed important for socialization of their adolescent children, adolescents themselves actively reinforce or decline parents’ attempts at socialization, in part through their own thoughts, beliefs, and the expectations they have for their parents (Lerner et al. 2002). In other words, adolescents could share information because they believe that parents have the right to know (Rote and Smetana 2016) or because they expect that their parents will (be able to) provide support and guidance (Chaparro and Grusec 2015). Then again, parents would need to do something with that information in order to provide the support and guidance that their adolescents need and, in that sense pave the way for positive developmental outcomes for their adolescent children. Thus, another parenting factor could serve as a mediating mechanism in the links between adolescent communication efforts and their psychosocial outcomes. We believe that this idea should be paid more attention in future studies.

Although not expected, the negative link from adolescent emotional problems to adolescent disclosure was moderated by the family’s emotional climate, showing that adolescent emotional problems were predictive of lower disclosure within the context of a family’s negative emotional climate. Indeed, studies suggest that adolescents who experience internalizing problems tend to report lower parenting quality (Johnson and Greenberg 2013). As close relationships between parents and children generally bolster youth resilience and self-regulatory skills (Boldt et al. 2020), adolescents living within a context of a family’s poor emotional climate could have trouble finding adequate models for effective emotion regulation and coping with emotional distress. As experience of emotional problems is correlated with feelings of shame (Tangney et al. 2007), the feeling characterized by self-consciousness and a perceived sense of insufficiency, adolescents who do not perceive themselves to have supportive and warm relationships with parents, would rather hide their feelings (Eisenberg 2000), than seek support from their parents, which in turn leaves parents less engaged in their adolescents’ lives. This is worrying because this vicious circle risks jeopardizing adolescent psychological development as well as the bond between parents and their adolescent children. Thus, nurturing a positive emotional climate in the family could be important not only for adolescent psychosocial development but also for parent-adolescent communication and parents’ involvement in adolescents’ lives.

### Limitations and Future Directions

This study has limitations that need attention. First, the measures of family emotional climate do not capture the full extent of the highly complex concept of family emotional climate, as defined for instance by Halberstadt and Eaton (2002); neither do they necessarily concern the entire family unit. Much remains unknown about the expression and communication of emotions in the family context. Second, there are concerns with common method variance for adolescent reports, including measures of parental behavioral control and adolescents’ feelings of being overly controlled that are slightly similar. As parents and adolescents tend to perceive their relationship as well as parenting behaviors somewhat differently (de Los Reyes 2011), future studies should also include parents’ reports of parent-adolescent communication and family emotional climate. Third, the adolescent conduct problems scale provided poor internal reliability on both timepoints. Although the scale generally has less than acceptable Cronbach’s alpha scorings (<0.70) the adolescent conduct problems scale has good test-retest reliability and predictive validity (Lundh et al. 2008). Moreover, the CFA of the scale provided acceptable goodness of fit on both time points (T1: CFI/TLI = 0.98/0.95 RMSEA <0.04; T2: CFI/TLI = 0.95/0.91 RMSEA <0.06). Fourth, there is item overlap on the measures of adolescent conduct problems and delinquency concerning stealing. Excluding the item, however, did not significantly alter the results. Fifth, because of the lack of socioeconomic diversity in the sample, it is uncertain whether the results would apply to families with low socioeconomic status as parenting practices and their effect on adolescent developmental outcomes might differ in different socioeconomic contexts (Rekker et al. 2017). Finally, our measures were operationalized without separating mothers’ and fathers’ parenting behaviors or adolescent communication with mothers and fathers. Parenting effects from mothers and fathers may however vary by adolescent gender (McKinney and Renk 2008). To obtain a more robust picture of parent-adolescent communication as well as the family’s emotional climate, exploring communication and relationships between different parent-adolescent dyads is warranted.

## Conclusions and Implications

Our findings suggest that the effect of parent-driven communication efforts (i.e. parental solicitation and behavioral control) on adolescent psychosocial functioning is not isolated from the context of the family’s emotional climate. When used within the context of a family’s positive emotional climate, parents’ questions and requests for information are protective against adolescent externalizing problems over time. On the other hand, parents’ rules and behavioral expectations are disadvantageous for adolescent behavioral development when used within the context of a family’s poor emotional climate. In other words, when the family’s emotional climate is positive, parental actions can result in advantageous developmental outcomes in adolescents. In contrast, when a family’s emotional climate is poor, the effect of parental actions is questionable or even disadvantageous to adolescent developmental outcomes. Such findings contribute to the previous research by adding specificity to the previously established role of parent-adolescent communication in adolescent psychosocial development.

Our findings indicate further that adolescent disclosure is protective of adolescent psychosocial functioning, while adolescent secrecy is linked to higher levels of adolescent psychosocial problems and a lower sense of wellbeing over time. Adolescent-driven communication efforts have an effect on adolescent developmental outcomes regardless of the state of adolescents’ perceptions of the family’s emotional climate. As adolescent disclosure and secrecy are adolescent-induced mechanisms, we suggest that other cognitive processes, such as adolescent beliefs and expectations (Chaparro and Grusec 2015) for their parents could in part explain links between adolescent-driven communication efforts and adolescent psychosocial functioning.

The findings in the current study underpin the importance of considering the context of a family’s emotional climate when providing recommendations to parents and in parenting programs. Put simply, prior to teaching specific parenting strategies such as setting clear rules, professionals should be offering strategies to promote the family’s emotional climate, including emotional connectedness between parents and their adolescent children. This could particularly be important in families with unstable home environments. At the same time, it should be noted that more studies are needed to identify predictors of a family’s emotional climate in families with adolescents. It is likely that the family’s emotional climate is established during earlier stages of the family life cycle (Rodgers and White 1993). If so, then interventions aimed at promoting the family’s emotional climate should target families before the children become adolescents. Finally, enhancing adolescent disclosure and reducing adolescent secrecy should be emphasized in the prevention of adolescent psychosocial problems and functioning.

## Data Availability

The datasets generated and analyzed during the current study are not publicly available but are available from the corresponding author on reasonable request.
